# Two Cases of Lameness in the Region of the Femero-tibial Articulation1Presented at the clinic for post-graduates at the McKillip Veterinary College.

**Published:** 1898-02

**Authors:** W. E. A. Wyman

**Affiliations:** M’killip Veterinary College, Pro Tempore; Clawson Agricultural College, S. C.


### TWO CASES OF LAMENESS IN THE REGION OF THE FEMORO-TIBIAL ARTICULATION.[1]

W. E. A. Wyman, V.S.,
M'KILLIP VETERINARY COLLEGE, PRO TEMPORE; CLAWSON AGRICULTURAL COLLEGE, S. C.

Case I. *Lameness due to tumor-formation in the triceps femoris.* —The animal ran away and received a hard blow over the triceps femoris, about four to seven centimetres above the articulation;

[1] Presented at the clinic for post-graduates at the McKillip Veterinary College.

although slightly lame the owner continued to work the animal. Lameness eventually became so marked as to seriously interfere with locomotion.

On inspection, in the standing posture, the whole leg is abducted, the toe turned out a little, and the region of the stifle appears swollen. While walking there is marked swinging-leg lameness with abduction. The stifle- and hock-joints are flexed imperfectly, the animal refraining from flexing them as much as possible. The motion is mainly confined to the coxo-femoral and phalangeal articulations. When walking beyond a certain pace the toe drags and the animal stumbles.

On palpation, passive flexion of the stifle-joint is difficult, the tumor mechanically opposing flexion, which at the same time is painful. Palpation reveals a globular, hard tumor about ten centimetres in diameter and immovable. It involves the triceps femoris and large adductor of the thigh.

The fact that the animal made a good recovery in about six weeks makes it especially interesting. At that time palpation did not reveal anything abnormal, while inspection showed a slight abduction of the limb during trotting.

The treatment consisted of bilateral setoning.

CASE II. *Bilateral gonitis.*—Heavy draught-horse employed in hauling lumber. Lame at first in the near hind leg; fourteen days later the right leg became involved. The animal stood up most of the time, experiencing more or less difficulty in rising when down. Shortly after lameness was manifested, appetite became impaired, the animal rapidly losing flesh.

On inspection, while standing still, the animal continuously flexes the leg, elevating it until there is danger of falling over. During intervals of rest the joint below the patellæ appears greatly distended. The emaciated animal seems in pain, the abdomen visibly tucked up. When walking the legs are moved stiffly, in other words, there is decided swinging-leg lameness; the toe is apt to trip, and is worn. Whenever the animal turns sharply the excessive spasmodic flexion of the limb reminds one forcibly of stringhalt.

On palpation, passive flexion, while free enough, was actively opposed by the patient. The capsular ligament below the patellæ feels tense, no pain being produced by manipulation.

The animal, rapidly becoming useless, was killed. The postmortem examination, made by the writer, revealed some blood-clots, the origin of which was not detected. There was an excess of thick synovia. The usual pathological changes in the synovial membranes, cartilage, and bone were wanting.

The rapidly fatal termination of this case is of special interest, as such lamenesses, while practically incurable, often cover months if not years before the animals become useless.

